# 

**Published:** 1849-07-01

**Authors:** 


### Notice to Correspondents.

We have received numerous Reports of county asylums, pamphlets, and books, all of which will be noticed in our next number. We have unavoidably been obliged to postpone our usual Notice to Correspondents, owing to a sudden press of matter.

Parties wishing to dispose of copies of No. II. of the Journal, or to exchange them for recent numbers, are requested to intimate their wish to the publisher.

